# Elevated blood pressure level based on 2017 ACC/AHA guideline in relation to stroke risk in rural areas of Liaoning province

**DOI:** 10.1186/s12872-019-1197-x

**Published:** 2019-11-20

**Authors:** Yanxia Xie, Mingfeng Ma, Zhao Li, Xiaofan Guo, Guozhe Sun, Zhaoqing Sun, Jia Zheng, Yingxian Sun, Liqiang Zheng

**Affiliations:** 1 Department of Clinical Epidemiology, Library, Department of Health Policy and Hospital Management, Shengjing Hospital of China Medical University, Shenyang, 110004 People’s Republic of China; 2 Department of Cardiology, Zhuhai People’s Hospital, Zhuhai Hospital Affiliated with Jinan University, Zhuhai, 519000 People’s Republic of China; 3 Department of Cardiology, the First Affiliated Hospital of China Medical University, Shenyang, 110001 People’s Republic of China; 4 Department of Cardiology, Shengjing Hospital of China Medical University, Shenyang, 110004 People’s Republic of China

**Keywords:** Blood pressure, Stroke, Incidence, Prospective study, Rural area

## Abstract

**Background:**

The new ACC/AHA hypertension guideline lower the definition of hypertension from 140/90 mmHg to 130/80 mmHg and eliminate the category of prehypertension thus increasing the prevalence of hypertension. A purpose of this study is to explore the applicability of the new guidelines in rural China.

**Methods:**

In total, 3229 participants aged ≥35 years and free of stroke at baseline were followed for up to 4.8 years during 2012 to 2017 in a rural community-based prospective cohort study of Xifeng County. The hazard ratio (HR) and 95% Confidence interval (CI) of different blood pressure (BP) levels for risk of incident stroke were analyzed by multivariable Cox proportional hazard models.

**Results:**

During the follow-up, 81 new strokes occurred among the 3229 participants. Compared with normal BP (Systolic BP (SBP)<120 mmHg and Diastolic BP (DBP)<80 mmHg), stage 2 hypertension (SBP ≥ 140 mmHg or DBP ≥ 90 mmHg) had approximately 2.1 greater risks for stroke (HR: 2.10, 95% CI: 1.13 to 3.91, *P* = 0.020). However, there was no significant association between elevated (SBP:120-129 mmHg and DBP<80 mmHg), stage1 hypertension (SBP:130-139 mmHg or DBP:80-89 mmHg) and stroke incidence (HR: 0.93, 95% CI: 0.33 to 2.61, *P* = 0.888; HR: 0.96, 95% CI: 0.46 to 2.02, *P* = 0.920, respectively). An increase of the SBP by 1-SD increases the risk for stroke by 56% (HR: 1.56, 95%CI: 1.29 to 1.88, *P* < 0.001). An increase of the SBP by 20 mmHg increases the risk for stroke by 51% (HR: 1.51, 95%CI: 1.27 to 1.80, *P* < 0.001).

**Conclusions:**

Compared with normal BP, the stage 2 hypertension based on 2017 ACC/AHA guideline significantly increases the risk of stroke incidence, but this association was not observed between elevated, stage1 hypertension and stroke incidence in Chinese rural adults.

## Background

In the past decade, the relationship between blood pressure (BP) and stroke has been well documented [1–4]. In the United States, hypertension caused more deaths from cardiovascular disease (CVD) than any other CVD risk factor that is changeable, making it become the second preventable cause of death after smoking [1]. The national health and nutrition survey (NHANES) study shows that more than 50% of patients who died from coronary heart disease (CHD) or stroke are from high blood pressure [2]. The risk of Atherosclerosis Risk in Communities (ARIC) study indicates that 25% of cardiovascular events (CHD, coronary artery disease reconstruction, stroke or heart failure) are derived from hypertension [3]. The death of cardiac cerebral vascular disease is the first cause of death in China. Among them, stroke is the first cause of death [4]. Many studies have also reported that China has one of the highest incidences of stroke worldwide [5–8].

However, newly issued on November 13, 2017, the American College of Cardiology / American Heart Association (ACC/AHA) guidelines revised the hypertension definition to 130/80 mmHg and increasing the prevalence of hypertension. BP was divided into the normal (Systolic BP (SBP)<120 mmHg and Diastolic BP (DBP)<80 mmHg), elevated (SBP:120-129 mmHg and DBP<80 mmHg), stage1 hypertension (SBP:130-139 mmHg or DBP:80-89 mmHg) and stage 2 hypertension (SBP ≥ 140 mmHg or DBP ≥ 90 mmHg). Similarly, if China follows the guideline of the United States, more people will become hypertensive patients, especially in the rural population, many previous studies conducted by our team also have shown that the prevalence of hypertension is high in rural population in Liaoning [9–11].

Therefore, this study used the 2017 AHA/ACC hypertension guideline to define BP level and observe the relationship between new BP levels and the risk of stroke incidence through a rural community-based prospective cohort study in order to explore the applicability of the new guidelines in rural China.

## Methods

### Study population

This study was based on a rural community-based prospective cohort study of Xifeng County, which is located in northeast China. From June 2012 to August 2012, 4157 Chinese rural participants aged ≥35 years from 2 of the 19 towns were included by cluster random sampling (2 towns, Anmin and Helong). Individuals who are pregnant, malignancies or mental disorders were excluded from this study. The study was approved by Ethics Committee of China Medical University (Shenyang, China)(Num. AF-SOP-07-1. 0–01). All procedures were conducted in accordance with the ethical standards of this committee. All participants received written consent after learning the objectives, benefits and medical details of the study and the confidentiality agreement regarding personal information.

**Fig. 1:**
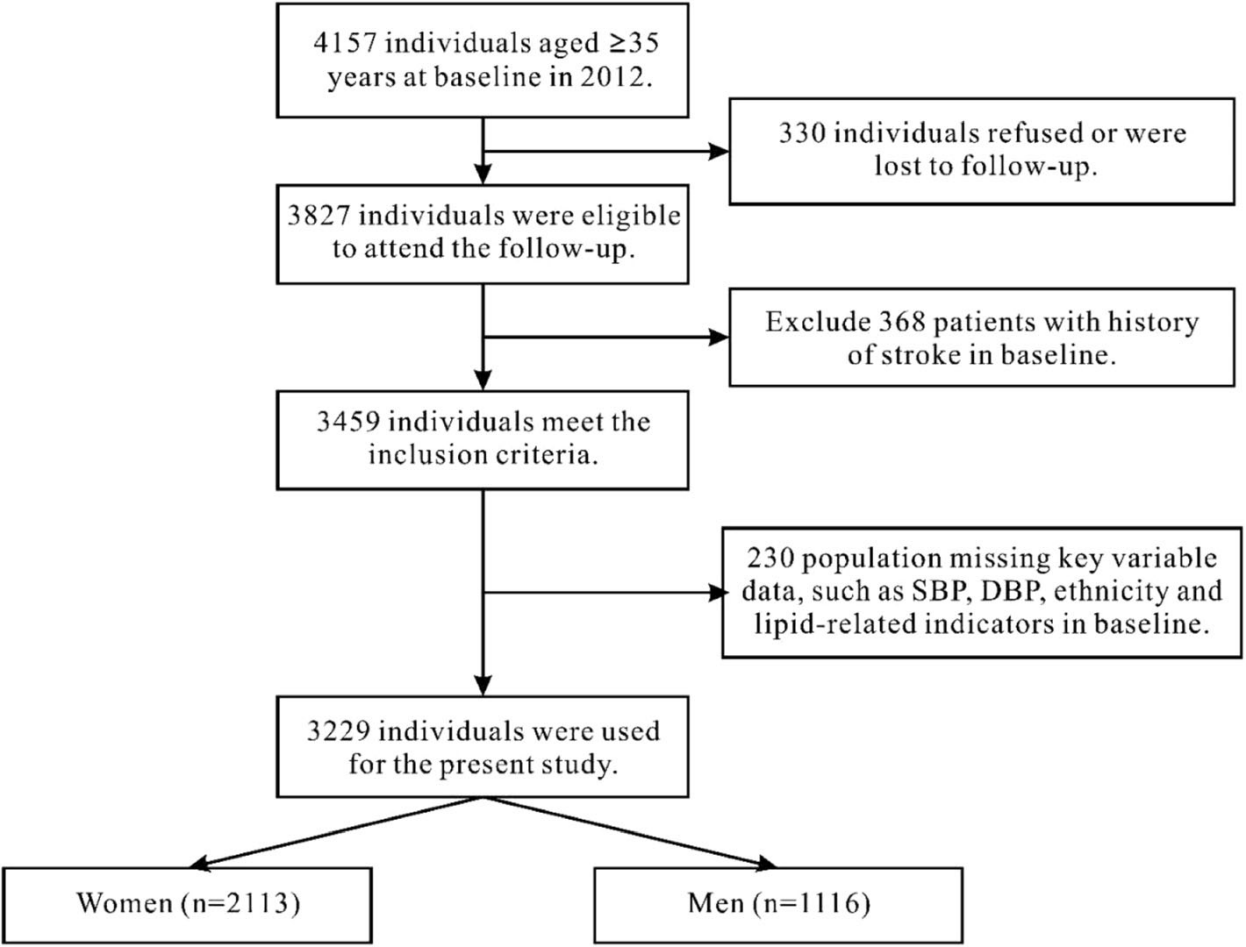
Flow chart of participant recruitment and derivation of the population used in the final analysis. Abbreviation: SBP, systolic blood pressure; DBP, diastolic blood pressure

All study subjects were invited to return for follow-up from June 2017 to September 2017, 330 individuals refused or were lost to follow-up and 3827 (92.06%) participants (or their guardians) agreed and completed the follow-up study. In this study, we analyzed the baseline and follow-up data, and only the participants provided complete data on the variables were analyzed in the study. The inclusion process of subjects is shown in Fig. 1**.**

### Stroke and BP level assessment

In our study, on the basis of the WHO multi-national monitoring of the trend of cardiovascular disease and the decision factor (MONICA) standard [12], the stroke event is defined as a local (or global) brain dysfunction that continues the rapid growth of the > 24 h, with no apparent non-vascular causes. The definition included patients presenting with clinical signs and symptoms of subarachnoid hemorrhage, intracerebral hemorrhage, thrombosis. Transient ischemic attacks (TIA) and silent brain infarctions (cases without clinical signs or symptoms) were not included, neither were events associated with trauma, hematologic disorders, or malignancy. All information was independently reviewed by the endpoint evaluation committee and its members were unaware of the baseline risk factors for the study participants.

### BP measurement and definition

According to the American heart association protocol, the BP was measured three times at least 2 min after a rest of at least 5 min using a standardized automatic electronic blood pressure measuring instrument (HEM − 907), which has been confirmed by British hypertensive association [13]. Participants were advised to avoid sports and alcohol for at least 30 min before they were measured. During the measurement, the participants’ arms were supported at the heart level. The average of three BP was used for the final analysis and evaluation.

According to the American Heart Association (AHA) published its 2017 hypertension clinical practice guidelines, BP was divided into the normal (SBP<120 mmHg and DBP<80 mmHg), elevated (SBP:120–129 mmHg and DBP<80 mmHg), stage1 hypertension (SBP:130–139 mmHg or DBP:80–89 mmHg) and stage2 hypertension (SBP:≥140 mmHg or DBP:≥90 mmHg) [14].

### Data collection and measurements at baseline

Detailed methods have been described previously [15]. The data was conducted by face-to-face with cardiologists and trained nurses using standard questionnaires during a single clinic visit. Prior to the investigation, we invited all qualified investigators to participate in an organized training course. The training included the purpose of this study, how to manage the questionnaire, the standard measurement method, the importance of standardization and the research procedure. The training was followed by rigorous test, and only those who scored high on the test were allowed to act as investigators. Our inspector provided further instructions and support when collecting data.

A standardized questionnaire, had been described in previously literature [7, 9, 11, 15, 16], was used to investigate the demographic characteristics, lifestyle risk factors, family income, history of heart disease and any drugs used in the 2 weeks before the survey. Currently smoking was defined as at least one cigarette per day and for at least 1 year [16]. Alcohol consumption was defined as the weekly consumption of beer, wine and hard liquor, converted into mL of alcohol. Current drinking was defined as more than 1 drink/day for women and more than 2 drinks/day for men during the last year [16]. Education levels were divided into primary or middle school, middle school, and high school or above. Labor strength is based on the classification of China physical labor intensity, divided into 4 levels**:** I (Light labor), II (Moderate labor), III (Heavy labor), IV (Extremely heavy labor). Diabetes was defined as a history of diabetes or fasting blood glucose (FPG) level ≥ 7.0 mmol/L [17]. The history of antihypertensive drugs was taken within 2 weeks of the patient’s self-report. The history of coronary heart disease (CHD) was defined as patient’s self-report. Weight and height were measured to the nearest 0.1 kg and 0.1 cm, respectively, with participants in light weight clothing and without shoes. Body mass index (BMI) was calculated as the participant’s weight in kilograms divided by their height in meters squared (kg/m^2^).

After a fasting of at least 12 h, all participants collected fasting blood samples. Then isolated from whole blood serum, and serum samples were frozen at − 20 °C for testing at a center, certified laboratory. In automatic analyzer (Olympus AU640 automatic analyzer, Olympus Corp., kobe, Japan), general blood biochemical indexes such as total cholesterol (TC), FPG, low-density lipoprotein cholesterol (LDL-C), high density lipoprotein cholesterol (HDL-C), triglyceride (TG), were analyzed. All laboratory equipment has been calibrated and samples are repeated with blind samples. Dyslipiemia was defined as TG ≥ 1.7 mmol/L, TC ≥ 5.2 mmol/L, LDL-C ≥ 3.4 mmol/L or HDL-C < 1.0 mmol/L [18].

### Statistical analysis

Descriptive statistics were calculated for all the variables, including continuous variables (expressed as the mean values and standard error) and categorical variables (expressed as numbers and proportions). The difference of baseline characteristics between different sex were evaluated using the student’ t test or the χ^2^-test, as appropriate.

To evaluate stroke risk associated with BP level, participants were stratified by BP, which was according to the 2017 ACC/AHA classification criteria: normal, elevated, stage1 hypertension and stage 2 hypertension. Cox proportional hazards model (Forward Stepwise) was used to identify independent associations between different BP level or an increment of 1- standard deviation (SD) /20 mmHg in SBP/DBP and stroke incidence. All the variables included in the analysis start including: age, sex, ethnicity, education level, current smoking, current drinking, BMI, labor strength, dyslipidemia, diabetes, atrial fibrillation, coronary heart disease and antihypertensive medication. Hazard Ratios (HR) and the corresponding 95% confidence interval (CI) were calculated, with the normal BP level (SBP/DBP <120/<80 mmHg) as the reference. In addition, sensitivity analyses were performed after excluding participants who were taking antihypertensive medications. Furthermore, an age group BP level and a sex BP level interaction were analyzed in Cox proportional hazards model.

The incidence rate was denoted by case load/100,000 person-years. χ^2^ test was used to test the rate difference between sex.

All the statistical analyses were performed using SPSS version 20.0 software, and a 2-sided value of *P* less than 0.05 were considered to be statistically significant.

## Results

### Baseline characteristics of the study population

A total of 3229 rural Chinese adults (65.4% women; mean age, 55.6 ± 10.2 years), with a median follow-up period of 4.8 years, were eligible for the present study. Table 1 shows the baseline characteristics of all participants. The mean SBP and DBP of the present study participants are 129.4 ± 21.2 mmHg, 82.2 ± 12.4 mmHg, respectively. There were statistical differences in age, education level, current smoking, current drinking, labor strength, BMI, atrial fibrillation, history of CHD, DBP and BP level among different sex. Overall, the proportion of normal BP level, elevated BP level, stage 1 hypertension, and stage 2 hypertension were 26.9, 8.8, 29.1, and 35.2%, respectively. Only a total of 3.4% of the population took antihypertensive drugs. According to the 2017 ACC /AHA guideline, hypertension prevalence rate was 64.5%.

**Table 1:** Baseline characteristics of study population (*N* = 3229)

	Women (*n* = 2113)	Men (*n* = 1116)	*P* Value	Total *N* = 3229
Age (years)	54.56 ± 0.21	57.58 ± 0.32	0.014	55.60 ± 0.18
Ethnicity, %				
Han	52.3	55.6		53.5
Manchu	46.0	43.1		45.0
Mongolian	1.7	1.3	0.189	1.5
Education level, %				
Primary school or below	73.8	59.1		68.7
Middle school	22.0	30.0		24.7
High school or above	4.2	10.9	<0.001	6.5
Current smoking, %	21.4	55.4	<0.001	33.1
Current drinking, %	4.7	43.9	<0.001	18.2
BMI (kg/m^2^)	23.39 ± 0.08	22.93 ± 0.10	<0.001	23.23 ± 0.06
Labor strength				
I	21.8	17.3		20.2
II	23.7	18.6		22.0
III	44.1	43.4		43.8
IV	10.4	20.7	<0.001	14.0
Dyslipidemia, %	64.9	61.7	0.073	63.8
Diabetes, %	9.7	10.2	0.673	9.9
Atrial fibrillation, %	4.7	2.9	0.013	4.1
SBP (mmHg)	128.19 ± 0.47	132.76 ± 0.62	0.157	129.40 ± 0.37
DBP (mmHg)	80.89 ± 0.25	84.48 ± 0.36	0.044	82.15 ± 0.22
BP level^a^				
Normal	31.2	18.8		26.9
Elevated	9.0	8.3		8.8
Stage 1	28.2	31.0		29.2
Stage 2	31.6	41.8	<0.001	35.2
History of CHD, %	11.5	7.7	0.001	10.2
Antihypertensive medications, %	3.7	2.9	0.916	3.4

Abbreviations: *BMI* body mass index, *TC* total cholesterol, *TG* triglyceride; *LDL-C* low density lipoprotein cholesterol; *HDL-C* high-density lipoprotein cholesterol; *CHD* Coronary heart disease, *SBP* systolic blood pressure, *DBP* diastolic blood pressure. P for category from chi-square; for continuous from student’ t test. ^a^ BP level was defined by 2017ACC/AHA hypertension guideline

### Multivariate cox regression analyses for BP and stroke

Table 2 presents the multivariate Cox regression analyses for the risk of stroke in incidence relation to different BP levels (the specific global *p*-value is 0.011). Stage 2 hypertension patients had approximately 2.10 greater hazard for stroke (HR: 2.10, 95% CI: 1.13 to 3.91, *P* = 0.020) compared with the normal BP. Overall, there was no significant association between elevated BP and stroke incidence (HR: 0.93, 95% CI: 0.33 to 2.61, *P* = 0.888). And our study also showed that there was no significant association between stage1 hypertension and stroke incidence (HR: 0.96, 95% CI: 0.46 to 2.02, *P* = 0.920).

**Table 2:** Cox regression analyses for association between BP level and stroke in the total population(*N* = 3229) (Forward Stepwise)

Variable	HR	95%CI	*P*-Value
Model 1			
BP level			0.011^a^
normal	reference		
elevated	0.93	0.33–2.61	0.888
stage1	0.96	0.46–2.02	0.920
stage2	2.10	1.13–3.91	0.020
Age	1.06	1.03–1.08	< 0.001
Current drinking	1.76	1.09–2.84	0.021
Model 2			
SBP (per SD)	1.56	1.29–1.88	< 0.001
Age	1.05	1.03–1.07	< 0.001
Current drinking	1.80	1.12–2.90	0.016
Model 3			
SBP (per 20 mmHg)	1.51	1.27–1.80	< 0.001
Age	1.05	1.03–1.07	< 0.001
Current drinking	1.80	1.12–2.90	0.016

Abbreviations: *HR* Hazard Ratios, *CI* confidence interval

All the variables included in the analysis start including age, sex, ethnicity, education level, current smoking, current drinking, BMI, labor strength, dyslipidemia, diabetes, atrial fibrillation, coronary heart disease and antihypertensive medication

^a^represent the correct global *p*-value for the BP categorical variable included in the Cox model

Model 1: the independent variable is BP level

Model 2: the independent variable is SBP (per SD)/DBP (per SD)

Model 3: the independent variable is SBP (per 20 mmHg)/DBP (per 20 mmHg)

We further analyzed HRs for stroke incidence (Table 2) associated with a 1-SD or 20 mmHg increase of SBP and DBP. An increase of the SBP by 1-SD increases the hazard for stroke by 56% (HR: 1.56, 95%CI: 1.29 to 1.88, *P* < 0.001). An increase of the SBP by 20 mmHg increases the hazard for stroke by 51% (HR: 1.51, 95%CI: 1.27 to 1.80, *P* < 0.001). However, no statistical significance was found between a 1-SD or 20 mmHg increase of DBP and stroke incidence.

In addition, similar results were found after excluding participants on antihypertensive treatments (*n* = 3118) (the specific global *p*-value is 0.047) (Table 3). Furthermore, we did not find there is any effect medication by sex or age group in the multivariable models (*P* = 0.644, *P* = 0.961, respectively).

**Table 3:** Cox regression analyses for association between BP level and stroke in population without antihypertensive treatments (*n* = 3118) (Forward Stepwise)

Variable	HR	95%CI	*P*-Value
Model 1			
BP level			0.047^a^
normal	reference		
elevated	0.90	0.32–2.54	0.844
stage1	0.98	0.46–2.05	0.947
stage2	1.90	1.01–3.58	0.047
Age	1.06	1.03–1.08	< 0.001
Current drinking	1.86	1.13–3.04	0.014
Model 2			
SBP (per SD)	1.51	1.24–1.84	< 0.001
Age	1.05	1.03–1.08	< 0.001
Current drinking	1.88	1.15–3.08	0.012
Model 3			
SBP (per 20 mmHg)	1.46	1.21–1.76	< 0.001
Age	1.05	1.03–1.07	< 0.001
Current drinking	1.88	1.15–3.08	0.012

Abbreviations: *HR* Hazard Ratios, *CI* confidence interval

All the variables included in the analysis start including age, sex, ethnicity, education level, current smoking, current drinking, BMI, labor strength, dyslipidemia, diabetes, atrial fibrillation, coronary heart disease and antihypertensive medication

^a^represent the correct global *p*-value for the BP categorical variable included in the Cox model

Model 1: the independent variable is BP level

Model 2: the independent variable is SBP (per SD)/DBP (per SD)

Model 3: the independent variable is SBP (per 20 mmHg)/DBP (per 20 mmHg)

### Incidence rate of stroke

**Fig. 2:**
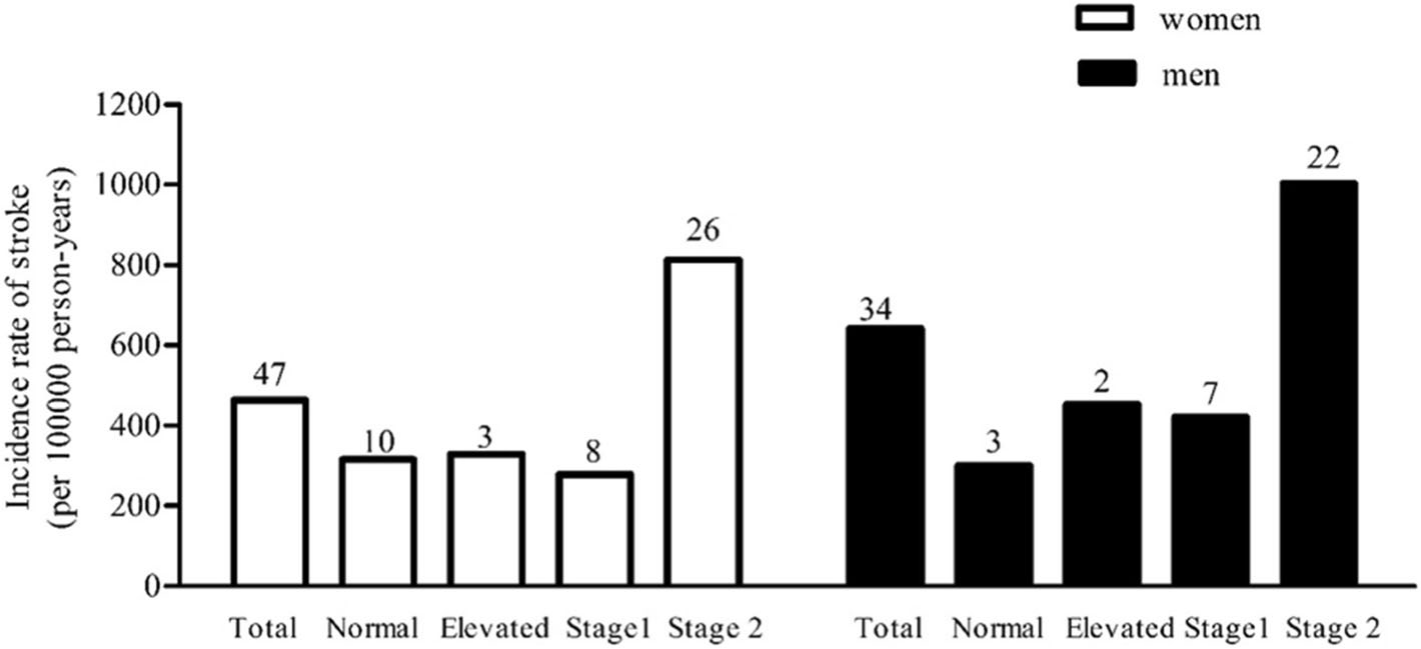
Incidence rate of stroke (per 100,000 person-years) in different BP level between women and men. Abbreviation: the digital on top of the bars represent the number of stroke events

During follow-up, 81 (2.5%) non-stroke individuals at baseline developed incident stroke, and the median follow-up year is 4.8. Overall, the incidence of stroke is on the rise across the four BP level: 311 per 100,000 person-years participants with normal BP, 367 per 100,000 person-years with elevated BP, 330 per 100,000 person-years participants with stage 1 hypertension and 889 per 100,000 person-years participants with stage 2 hypertension, respectively. Compare with other BP level, the higher incidence of stroke was obviously observed in stage 2 BP level among both women and men groups (813 per 100,000 person-years and 1002 per 100,000 person-years, respectively) (Fig. 2). In addition, no statistical significance was found among sex for incident stroke difference (*P* = 0.157).

## Discussion

The present study, with a relatively successful (> 90%) follow-up, revealed that the 2017 ACC/AHA stage 2 hypertension definition significant increases the risk of stroke incidence, but this association was not observed between elevated, stage1 hypertension and stroke incidence. And increment of 1-SD or 20 mmHg in SBP is associated with risk of stroke incidence. Furthermore, the rates of hypertension treatment and incident of stroke are disproportionately and unacceptably low among rural Chinese adults.

BP is a strong determinant risk factor of ischemic stroke and intracranial hemorrhage [19]. The present study showed that the Stage 2 BP level was associated with an approximately 2-fold greater risk for stroke. This result is consistent with most previous studies [20, 21]. According to a recent meta-analysis, BP control < 150/90 mmHg and low to moderate intensity(≤140/ 85 mmHg) was associated with significant decrease in stroke (RR, 0.74; 95% CI, 0.65–0.84; RR, 0.79, 95% CI 0.59–0.99, respectively) [20]. The multivariate-adjusted HR (95% CI) of stroke for non-hypertension/drinkers, hypertension/non-drinkers and hypertension/drinkers were 1.03 (0.48–2.22), 2.64 (1.45–4.81) and 2.89 (1.55–5.39), respectively, compared with non-hypertension/non-drinkers [21]. In a meta-analysis of clinical trials, antihypertensive therapy was associated with an average reduction in stroke incidence of 41% (95% CI, 33 to 48%), a reduction in SBP of 10 mmHg, or a DBP of 5 mmHg [22]. The combined results of SPRINT and ACCORD showed that the strengthen BP control (< 120 mmHg) compared with standard BP treatment level (< 140 mmHg) resulted in a significant reduction in stroke risk (RR, 0.75; 95% confidence interval: 0.58–0.97) [23]. In recent years, more and more studies have pointed out the risk of cardiovascular disease increases from normal BP to elevated BP and stage1 hypertension [24–26]. Many of these meta-analyses, the HR for stroke were between 1.5 and 2.0 for the comparison of SBP/DBP of 130–139/85–89 mmHg versus < 120/80 mmHg and between 1.1 and 1.5 for the comparison of SBP/DBP of 120–129/80–84 mmHg versus < 120/80 mmHg. However, the present study did not draw a similar conclusion. But, recently, a study from China reported that stage 1 hypertension on cardiovascular risk is evidenced in young and middle-aged Chinese adults, but not in those aged≥60 years [27] . This is a little similar to our finding but not exactly the same, which may be due to the differences age composition of the subjects or the relatively small sample size of our study.

Zengwu Wang, et al. showed that the prevalence of hypertension in rural residents according to the Chinese Hypertension Guidelines was 23.1% [28]. Our study indicated that the prevalence of hypertension according to the definitions from 2017 ACC/AHA guideline was 64.5%, which revealed that the 2017 ACC/AHA hypertension guideline will lead to a significant increase in the percentage of hypertension among Chinese rural adults. It is recommended that strengthen management and antihypertensive drug treatment to increase the control of hypertension in Chinese adults.

Our team previously reported the incidence of stroke in rural areas in northeast China from 2004 to 2010, of which men were 775.9 per 100,000 person-years and women were 435.6 per 100,000 person-years [29]. The good news is that the incidence of stroke in the rural areas of northeast China decreased slightly from 2012 to 2017, of which men were 641 per 100,000 person-years and women were 462 per 100,000 person-years. This is an affirmation of our stroke prevention work. However, in comparison with other ethnicity, the incidence of stroke in this area is still higher. One study showed that in 2005 the incidence of stroke among the White was 217 per 100,000 person-years, and the Black was 370 per 100,000 person-years [19]. These differences are likely to be the result of different Westernization lifestyles and eating habits. Accelerated epidemiological hypertension and low control rates may also be contributing factors.

In the present study, our study population was followed for a median of 4.8 years, and we provided updated information on the stroke and BP level, which is based on 2017 ACC/AHA Guideline for the Prevention, Detection, Evaluation and Management of High BP in Adults, among a general population from rural China. Although the representativeness of this study is more limited, the results provide unique and reliable information on the latest US guidelines on BP levels and stroke.

However, our study had some limitations that need to be considered. Firstly, BP measurement in baseline was just three times a day and it wasn’t three measurements on different days, which may induce a misclassification bias. Secondly, the relatively small number of stroke events meant that we had limited statistical power to detect the weak relationship between BP and stroke. Thirdly, our study cannot adjust for family history of CVD as we don’t collect this information in the baseline. Finally, the study sample only included rural adults in Liaoning province, we hope that it can be verified in larger population and more representative groups in the future.

## Conclusion

In conclusion, the stage 2 hypertension based on 2017 ACC/AHA guideline significantly increases the hazard of stroke incidence, but this association was not observed between elevated, stage1 hypertension and stroke incidence. Our study provides significant evidence of a link between the new guidelines’ BP level and the hazard of stroke incidence, and provides foundational data for future related studies, which are indispensable if application of the 2017 ACC/AHA hypertension guideline is to be considered in rural China even in China.

## Data Availability

The datasets generated and/or analyzed during the current study are available from the corresponding author on reasonable request.

## List of abbreviations

ACC/AHA: The American College of Cardiology / American Heart Association
ARIC: Atherosclerosis risk in communities
BMI: Body mass index
BP: Blood pressure
CHD: Coronary heart disease
CI: Confidence interval
CVD: Cardiovascular disease
DBP: Diastolic blood pressure
FPG: Fasting blood glucose
HDL-C: High density lipoprotein cholesterol
HR: Hazard ratio
LDL-C: Low-density lipoprotein cholesterol
NHANES: The national health and nutrition survey
SBP: Systolic blood pressure
TC: Total cholesterol
TG: Triglyceride
TIA: Transient ischemic attacks
